# UAV-Assisted Low-Consumption Time Synchronization Utilizing Cross-Technology Communication

**DOI:** 10.3390/s20185134

**Published:** 2020-09-09

**Authors:** Ziyi Tan, Xu Yang, Mingzhi Pang, Shouwan Gao, Ming Li, Pengpeng Chen

**Affiliations:** 1 School of Computer Science and Technology, China University of Mining and Technology, Xuzhou 221116, China; tanzy@cumt.edu.cn (Z.T.); yang_xu@cumt.edu.cn (X.Y.); MingzPang@cumt.edu.cn (M.P.); gaoshouwan@cumt.edu.cn (S.G.); lmgyw@cumt.edu.cn (M.L.); 2 China Mine Digitization Engineering Research Center, Ministry of Education, Xuzhou 221116, China

**Keywords:** time synchronization, cross-technology communication, energy consumption, UAV

## Abstract

Wireless sensor networks (WSNs) have been used in many fields due to its wide applicability. In this kind of network, each node is independent of each other and has its own local clock and communicates wirelessly. Time synchronization plays a vital role in WSNs and it can ensure accuracy requirements for coordination and data reliability. However, two key challenges exist in large-scale WSNs that are severe resource constraints overhead and multihop time synchronization errors. To address these issues, this paper proposes a novel unmanned aerial vehicle (UAV)-assisted low-consumption time synchronization algorithm based on cross-technology communication (CTC) for a large-scale WSN. This algorithm uses a UAV to send time synchronization data packets for calibration. Moreover, to ensure coverage and a high success rate for UAV data transmission, we use CTC for time synchronization. Without any relays, a high-power time synchronization packet can be sent by a UAV to achieve the time synchronization of low-power sensors. This algorithm can achieve accurate time synchronization with almost zero energy consumption for the sensor nodes. Finally, we implemented our algorithm with 30 low-power RF-CC2430 ZigBee nodes and a Da Jiang Innovations (DJI) M100 UAV on a 1 km highway and an indoor site. The results show that time synchronization can be achieved accurately with almost zero energy consumption for the sensor nodes, and the time synchronization error is less than 30 μs in 99% of cases.

## 1. Introduction

Time synchronization is one of the most fundamental and widely employed middle-ware services in wireless sensor networks (WSNs) [1,2,3,4]. Different nodes use their own local clock modules for timing and these local clock modules are implemented by crystal oscillators [2,3,5,6]. The frequencies of different crystal oscillators cannot be completely consistent. In addition, the local initial time of each node is not the same, even if the local initial time of some nodes are the same, the influence of different environmental factors such as electromagnetic waves, temperature, and humidity will cause clock deviation. Therefore, time synchronization operations are required to achieve time consistency between nodes. Accurate time synchronization can save communication energy, promote position accuracy, optimize monitoring range, and improve system security [7,8,9,10,11]. Previous research in this field mostly focused on how to do time synchronization for better clock accuracy, such as reference broadcast synchronization (RBS) [12,13], timing-sync protocol for sensor networks (TPSN) [14,15], and flooding time synchronization protocol (FTSP) [16,17,18,19]. However, the above algorithms have the following problems in large-scale sparse deployment networks: (1) The energy consumption; (2) Multihop time synchronization errors. Pottie and Kaiser [20] noted that the energy consumption of each sensor node sending 1 kb of data is equal to executing 3 million instructions when the transmission distance is longer than 100 m. In addition, achieving time synchronization with multiple hops in WSNs can lead to cumulative errors.

To address these issues, this paper proposes a novel unmanned aerial vehicle (UAV)-assisted low-consumption time synchronization algorithm called UAV-TS, which is based on cross-technology communication (CTC) for a large-scale wireless sensor network. The main idea is to apply a UAV to send time synchronization data packets. However, two challenges exist in a low-power WSN with large-scale deployment, where the communication distance is short and nodes face severe resource constraints. It is difficult to achieve universal and rapid time synchronization if the UAV uses traditional methods [21,22], whereas a high-power signal can quickly achieve time synchronization because of the greater communication distance and wider coverage [23,24,25,26]. Thus, we apply cross-technology communication [27] to time synchronization, which means that the UAV can achieve time synchronization by sending high-power data packets to simulate low-power data packets [28,29]. A WiFi transmitter carried by the UAV simulates the synchronization data of the wireless sensor node at the physical layer, so the synchronization data include the sequence number and timestamp required for time synchronization. The data are subsequently embedded into the payload of a high-power packet and broadcast periodically. Wireless sensor nodes can identify the data directly and achieve time synchronization according to the sequence number and timestamp. This means that sensor nodes can achieve precise time synchronization with almost zero energy consumption. Therefore, the main contributions of this paper are as follows:

- We present a novel UAV-assisted low-consumption time synchronization algorithm for a large-scale wireless sensor network that can achieve time synchronization with almost zero energy consumption.
- We provide a method of sending time synchronization data packets based on CTC. It uses a general commercial high-power WiFi device to broadcast time synchronization data packets to ensure that the UAV can achieve time synchronization universally and quickly.
- We implemented and evaluated the UAV-TS method with a sparse development sensor network (30 CC2430 ZigBee nodes) and a DJI M100 UAV (equipped with a WiFi device). Numerous experimental results demonstrate the effectiveness of our algorithm.

It is worth mentioning that although we are using the general commercial UAV, as long as the UAV carries high-power WiFi devices, our method can be applied in theory, which means that our method may be extended to some UAVs with constraints. For example, a deep learning environment using a radial basis function neural network (RBFNN) [30] and a nonlinear system controlled by a neural network (NN) and command filter-based [31]. The constraint requires further evaluation, we will not discuss it in detail here.

## 2. Related Work

### 2.1. Time Synchronization

Time synchronization is one of the most basic and widely-used technologies in wireless sensor networks. Time synchronization based on GPS is a commonly-used time synchronization method, which has high accuracy. However, the energy consumption of the node and the deployment cost of this solution is high. Another time synchronization protocol called precision time protocol (PTP) is proposed and defined in the IEEE 1588 standard [32]. This protocol is implemented at the application layer level and its precision is very high; however, PTP is only applicable to the client–server architecture. RBS is a time synchronization protocol commonly used in single-hop networks, which has low precision [12]. TPSN is a time synchronization protocol implemented in the Media Access Control (MAC) layer, it can be used in single-hop or multi-hop networks [14].

FTSP is the most widely-used time synchronization protocol to achieve communication between multiple nodes in wireless sensor networks [16,33]. It employs a broadcast mechanism to achieve time synchronization between nodes. In this method, nodes broadcast time synchronization information. Nodes within the synchronization range can receive synchronization information from other nodes to update their time synchronization information to achieve time synchronization.

### 2.2. Cross-Technology Communication

CTC is a new technology used to solve the communication between heterogeneous devices. Its characteristic is that heterogeneous wireless devices can directly exchange messages and data through a series of technical means. At present, the incompatibility between technologies and the asymmetry of equipment capacity are two major challenges of CTC. Commonly-used CTC technologies are mainly in the following two categories: packet-level CTC and physical-level CTC. The packet-level CTC mainly transmits information by using the modified packet as an information carrier. A CTC called Esense is a job that uses energy sampling to implement data transmission from WiFi to a ZigBee device [34]. It establishes an alphabet about the packet duration to transmit data, but this method is susceptible to interference and requires low noise in the environment. StripComm is a new type of CTC technology. It makes use of Manchester’s coding and proposes a coding mechanism for perceiving interference, which has strong anti-interference ability [25]. ZigFi focuses on the characteristics of channel state information (CSI) to implement Bluetooth Low Energy (BLE) to WiFi and ZigBee to WiFi communications, respectively [35]. FreeBee achieves CTC by changing the transmission time and embedding symbols into beacons, which can exchange data without a gateway [36]; however, FreeBee is limited by the beacon rate, resulting in its data rate is not high. Although packet-level CTC can solve the problem of communication between heterogeneous devices to a certain extent, the throughput of packet-level CTC is limited by the granularity of packet manipulation, and its size is measured in milliseconds.

The physical-level CTC focuses on the physical layer. According to the characteristics of the physical layer data packets, the data packets are modified to build collaboration between heterogeneous wireless devices on the physical layer to achieve CTC. Recently, a physical-level CTC called WEBee, which is the first CTC implemented at the physical-level [37]. It uses WiFi to send simulated ZigBee data packets and achieve high-performance CTC from WiFi to ZigBee. Our method is inspired by the FTSP and WEBee. It uses UAV to send simulated ZigBee data packets for time synchronization.

## 3. Algorithm Overview

**Figure 1:**
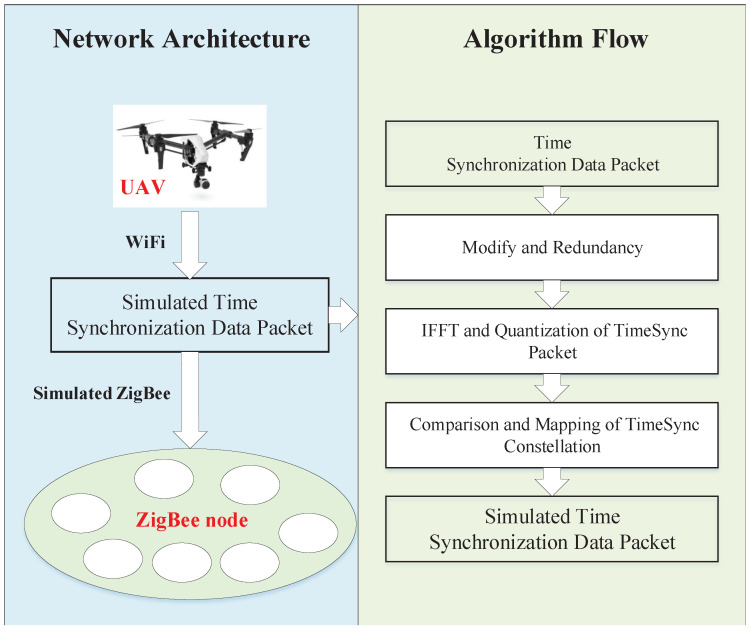
UAV-TS algorithm architecture.

UAV-TS algorithm architecture is shown in Figure 1. This algorithm consists of a transmitting module and a receiving module. The transmitting module is a WiFi device carried by a UAV. It can send simulated data packets embedded with corresponding beacons and timestamps, which can be recognized by ZigBee nodes. The receiving module is composed of many sparsely deployed ZigBee nodes to be synchronized. The sparse ZigBee nodes can receive the data packet and identify the timestamp and other information in it, thus achieving time synchronization.

The transmitting module sends WiFi data packets mainly including the following steps:

- The channel coding module encodes the data bits in the WiFi frame into redundant coded bits to enhance the robustness of the data. Map these coded bits to a series of constellation points through quadrature amplitude modulation (QAM). The QAM process formula is expressed as follows:
  (1) $$S_{MQAM}\left(t\right)=X\left(t\right)cos\omega_{c}t-Y\left(t\right)sin\omega_{c}t,$$
  where X(t) and Y(t) are two original signals and $cos\omega_{c}t$ and $sin\omega_{c}t$ are their corresponding quadrature modulation component.
- By using orthogonal frequency division multiplexing (OFDM) to insert pseudo-random pilot symbols, these constellation points are modulated into 48 data subcarriers. Meanwhile, pseudo-random pilot symbols are modulated into pilot subcarriers for channel estimation. The OFDM process formula is expressed as follows:
  (2) $$V\left(t\right)=\sum_{k=1}^{N_{c}}a_{k}cos\left(2\pi f_{k}t\right)+\sum_{k=1}^{N_{c}}b_{k}sin\left(2\pi f_{k}t\right)$$
  where $N_{c}$ is the number of subcarriers, $f_{k}$ is the preset carrier frequency interval, and $a_{k}$ and $b_{k}$ are different *k*-channel modulated subcarrier signals.
- After the subcarriers are processed by inverse fast Fourier transform (IFFT), all the subcarriers are combined and converted into a ZigBee time domain signal. As the following formula:
  (3) $$V\left(t\right)=\sum_{t=0}^{N-1}1/Nv\left(t\right)W_{N}^{-tk},k=0,\ldots ,N-1$$
- The ZigBee time domain signal is processed by a cyclic prefix module to form a cyclic prefix with an interval of 0.8 μs to eliminate inter-symbol interference and generate a simulated ZigBee signal.
- Finally, encapsulate the simulated ZigBee signal into the payload of the WiFi data packet and send the WiFi data packet.

Before sending the WiFi data packets, it needs to construct the simulated ZigBee time synchronization data packet. The construction of a simulated ZigBee time synchronization data packet is an important step. As shown on the right side of Figure 1, we obtain the ideal constellation point set $Q^{\prime }$ for WiFi transmission and modulation in advance, and $Q^{\prime }$ is as follows:

(4) $$Q^{\prime }\left(n\right)=\left\{\left(x_{1}^{\prime },y_{1}^{\prime }\right),\left(x_{2}^{\prime },y_{2}^{\prime }\right),\ldots \left(x_{n}^{\prime },y_{n}^{\prime }\right)\right\}$$

These are the preset constellation points. First, we modify the time synchronization data packet to enable better reception by the ZigBee nodes. The algorithm steps will be described in detail in the next section. Then, the time synchronization data packet is changed into a time domain waveform v(t), which can be recognized by the ZigBee devices by encapsulation at the MAC layer and the physical layer. Since the simulated ZigBee synchronization data packet is embedded in the payload of the WiFi data packet, the operation in the MAC layer is consistent with the general WiFi data packet transmission. After inverse fast Fourier transform and quantization, v(t) becomes another signal V(t), and the time domain waveform is mapped to a series of constellation points Q(n) as follows:

(5) $$V\left(t\right)=\sum_{t=0}^{N-1}v\left(t\right)W_{N}^{tk},k=0,\ldots ,N-1$$

(6) $$Q\left(n\right)=\left\{\left(x_{1},y_{1}\right),\left(x_{2},y_{2}\right),\ldots \left(x_{n},y_{n}\right)\right\}$$

where $W_{N}$ is the exponential factor and N−1 is the number of terms of the time domain waveform signal. We compare Q(n) and $Q^{\prime }$ and select the constellation point with the smallest total Euclidean distance ρ from Q(n) and $Q^{\prime }$, as shown in Formula (7).

(7) $$\rho =\sqrt{{\left(x_{1}^{\prime }-x_{1}\right)}^{2}+{\left(y_{1}^{\prime }-y_{1}\right)}^{2}}$$

Then, we can obtain the constellation point T(n) that can control the corresponding ZigBee data. According to T(n), the simulated time synchronization data packet can be obtained through the constellation mapping table and the coded bit mapping table.

Since the WiFi device is used as a transmitter, ZigBee can receive more synchronization information at the same time than the ZigBee node as the transmitter, which can achieve higher accuracy time synchronization in a shorter time. The advantages of this system are as follows:

- The transmission bandwidth of WiFi and ZigBee have overlapping parts, and this overlapping part can be used as a communication channel. At the same time, the transmission distance of WiFi can reach several hundred meters, which is much larger than the transmission distance of low-power ZigBee so it can cover a broader range. It means that using WiFi node as reference point has a certain theoretical basis, strong signal strength, and wide coverage.
- Modified the data packet format, so that the ZigBee receiver can more easily receive the simulated time synchronization data packet. Compared with the general ZigBee network, ZigBee nodes as receivers are only used to receive data and do not require extra energy to send data.

It is noteworthy that other further topics can be designed based on our topic. For example, ZigBee can be controlled to send data to a UAV by using CTC, so as to quickly collect data on the ZigBee side. In addition, UAV can send data to ZigBee nodes to achieve node localization. Since our main job is to achieve time synchronization, we only introduce the concept here and we will not elaborate on the scheme.

## 4. Algorithm Design

### 4.1. Analysis of Existing Problems of Physical-Level CTC

The traditional physical-level CTC has the following problems: (1) Hardware limitations and protocol incompatibility. It is necessary to construct a simulated ZigBee time synchronization data packet that can be recognized by the ZigBee node. Due to hardware limitations and incompatibility between different protocol standards, the simulated signal generated by a physical-level CTC system cannot perfectly match the required signal [38,39,40]. Therefore, compared with the standard ZigBee signal, the simulated signal will be partially distorted. (2) Signal and environmental interference. It is necessary to ensure that the timestamp in the data can be correctly identified and decoded as much as possible. The simulated time synchronization signal cannot completely match the required signal, and there is interference such as obstacles in the communication channel so the timestamp in the data may change [41,42]. For example, the quantized constellation point cannot completely coincide with the preset constellation point. Only the constellation point with the smallest Euclidean Distance can be selected for simulation, which will cause errors, as shown in the following formula:

(8) $$\int_{-T/2}^{T/2}{\left|\;u(t)-v(t)\right|}^{2}\;dt=T\sum_{k}{\left|\;U[k]-V[k]\right|}^{2}$$

where *T* is the period, u(t) is the initial time domain signal, v(t) is the time domain signal after quantization, and U[k] and V[k] are the time domain signals corresponding to the Fourier transform.

In the current physical CTC, these two problems exist objectively and cannot be completely avoided. Different algorithms can only be used to reduce the errors caused by these two problems. In order to solve these problems, the time synchronization data packet needs to be modified to make the ZigBee receiver receive the simulated data packet more easily.

### 4.2. Analysis of the FTSP Packet

Our algorithm needs to solve the following problems: (1) Hardware limitations and incompatibility between different protocol standards make it difficult to receive simulated data packets. Therefore, it is necessary to analyze different protocols and plan to adopt the best sending strategy according to the compatible parts of the protocol to obtain the best transmission. (2) Environmental interference in the communication channel, such as various obstacles, electromagnetic waves and bad weather, may cause the lack of key information such as the timestamp in the data packet, which causes the ZigBee nodes to consume a lot of energy to receive data and to be unable to obtain an accurate and complete timestamp for time synchronization. In order to solve these problems, we analyzed the time synchronization protocol of ZigBee and modified the original time synchronization data packet, which can effectively improve the packet reception rate.

**Figure 2:**
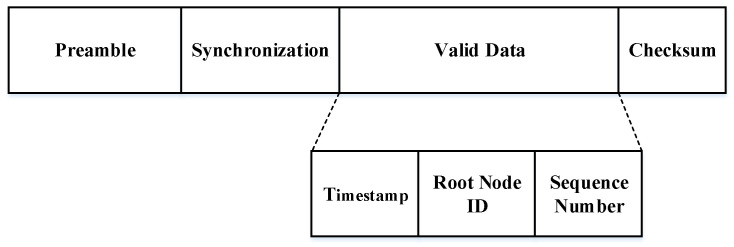
Flooding time synchronization protocol (FTSP) packet.

Our method is similar to the synchronization mechanism based on the send/receive protocol. To construct the simulated ZigBee time synchronization packet, we analyze the data packet format of the FTSP, a commonly-used time synchronization protocol. As shown in Figure 2, the data packet format of FTSP is mainly composed of four parts: The preamble is used by the ZigBee nodes to identify and receive ZigBee data packets; synchronization is used to align and determine whether the data are valid; valid data segments are used for synchronous calculation and the checksum.

The FTSP can achieve multihop time synchronization by root node communication [33,43,44]. It can dynamically select a root node as the clock source node according to the root node ID. The valid data segments of the synchronization information is divided into three parts: timestamp, root node ID, and sequence. The main workflow of the FTSP protocol is as follows: the root node broadcasts synchronization information, and every time new synchronization information is sent, the sequence of the synchronization information increases by 1. After that, the nodes within the receiving range can receive the synchronization information to start synchronization and become the new Root node. Finally, these new root nodes update the root node ID and sequence, broadcast the new synchronization information. Repeating the above steps to achieve time synchronization of the entire network.

### 4.3. Simulated Time Synchronization Data Packet

**Figure 3:**
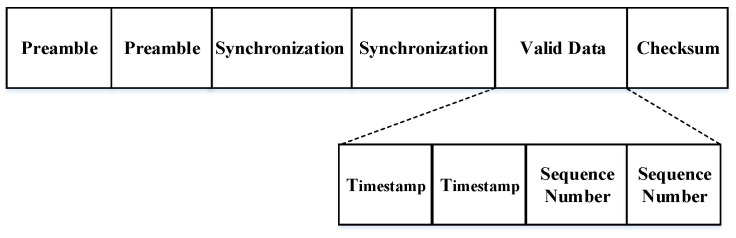
Modified synchronization packet.

Based on the format of the FTSP time synchronization packet and the characteristics of our design, we modify the data packet to ensure that our simulated time synchronization packet data can be recognized and received by the ZigBee nodes, as shown in Figure 3.

Our design uses WiFi to broadcast the simulated time synchronization data packet. WiFi has a longer transmission distance, and the time synchronization data packets need to be transmitted unidirectionally. The ZigBee nodes are only responsible for receiving the synchronization data. That is, only the WiFi device is used as a reference point that all nodes are synchronized to the WiFi device, so a root node ID is not needed in the data packet. At the same time, to reduce any deviation between the simulated time synchronization data packet and the original data packet, we introduce redundancy in the preamble and synchronization. In other words, we insert two identical preambles and synchronizations in the header of the data packet. This effectively improves the detection chances of the preamble and synchronization. Once the packet is identified, the other packet will be automatically discarded by the ZigBee node. The timestamp contains important information to achieve time synchronization. For the ith different transmission timestamps, we can record them as $T_{\left(sd,i\right)}$. The timestamp is processed similarly on the valid data to improve the successful recognition rate of the timestamp.

Since the working frequency bands of WiFi and ZigBee overlap, the ZigBee nodes can receive the WiFi data packet. Because the simulated RF waveform of the WiFi payload is similar to the RF waveform of the ZigBee signal [37], when the WiFi frame is transmitted through the WiFi Radio Frequency (RF) front end, the ZigBee device can ignore the WiFi header, preamble, and tail as noise, and the payload (i.e., the simulated time synchronization data packet) will be received successfully by the ZigBee node through ZigBee preamble detection. The received WiFi waveform is converted to baseband and digitized into in-phase and quadrature signals (I/Q) by using Analog to Digital Converter (ADC) for sampling. The phase shift p(n) between consecutive I/Q samples are used to demodulate the symbols, as shown in Formula (5):

(9) $$p\left(n\right)=\arctan\left(s(n)\times s*(n-1)\right)$$

where s(n) and s(n−1) are two consecutive samples. If the phase shift is less than $0^{{}^{\circ}}$, the chip is “0”; otherwise it is “1”. After collecting 32 chips, they are converted into ZigBee frames according to the chips. Finally, we can obtain the synchronization data.

### 4.4. Clock Deviation Modification

After designing the simulated time synchronization data packet, we need to correct the time offset to improve accuracy. The inconsistent frequencies of the crystal oscillators at each node cause different time rates, which expands the clock deviation between the nodes [7,45,46,47]. Therefore, it is necessary to perform regular time synchronization. We assume that the drift rate of clock *x* relative to the reference clock *y* is expressed as $S_{\left(y,x\right)}$, so the drift rate can be expressed by the following formula:

(10) $$S_{\left(y,x\right)}=\frac{d_{2}-d_{1}}{t_{y2}-t_{y1}}=\frac{\left(t_{y2}-t_{x2}\right)-\left(t_{y1}-t_{x2}\right)}{t_{y2}-t_{y1}}$$

where $t_{x1}$, $t_{x2}$, $t_{y1}$ and $t_{y2}$ are two consecutive unit times of *x* and *y* nodes, respectively. $d_{1}$ and $d_{2}$ are clock deviations of the corresponding unit times.

Our design is different from the general synchronization system, it uses the UAV to carry WiFi, and the coverage of WiFi is wide. Therefore, placing the WiFi device in different positions can be regarded as different reference points for performing linear regression to estimate the clock drift rate skew of the node, as shown in Formula (11).

(11) $$skew\left(n\right)=\frac{\sum_{i=n-m+1}^{n}\left(T_{\left(os,i\right)}-\bar{T_{os}}\right)\left(T_{\left(a_{v},i\right)}-\bar{T_{av}}\right)}{\sum_{i=n-m+1}^{n}{\left(T_{\left(av,i\right)}-\bar{T_{av}}\right)}^{2}}$$

(12) $$T_{\left(os,i\right)}=T_{\left(sd,i\right)}-T_{\left(av,i\right)}$$

where skew(n) is the clock drift rate obtained by linear regression on the linear regression table (The part which saving the useful time information for linear regression in ZigBee) for the *n*th time period, *n* is the number of synchronization periods and m is the number of reference points stored in the linear regression table. When the linear regression table obtains m reference points, the linear regression calculation can be started, so n≥m is required. $T_{\left(os,i\right)}$ is the clock offset obtained by the *i*th time reference point in the linear regression table, $T_{\left(sd,i\right)}$ is the transmit timestamp of the *i*th reference point in the linear regression table, and $T_{\left(av,i\right)}$ which generated by the ZigBee node after receiving the time synchronization data packet is the receive timestamp of the *i*th reference point in the linear regression table. $\bar{T_{os}}$ and $\bar{T_{av}}$ are the averages of the clock offset and received timestamp of the reference points in the linear regression table.

According to the clock drift rate obtained by Formulas (11) and (12), the formula for adjusting the *n*th time synchronization period is as follows:

(13) $$T_{gb}=T_{lc}+\bar{T_{os}}+skew\left(n\right)*\left(T_{lc}-\bar{T_{av}}\right)$$

where $T_{gb}$ is the global time after adjustment and $T_{lc}$ is the local time at the node. Using linear regression can effectively compensate for clock drift to improve accuracy.

To ensure the reliability of the system, we re-transmitted the simulated time-synchronized data packets. Reasonable retransmission can effectively improve the packet reception rate. For different retransmission times, we denote it as $T_{i}$. Although this measure reduces the throughput, it is acceptable for the time synchronization of the ZigBee nodes.

## 5. Algorithm Evaluation

### 5.1. Experimental Setup

**Figure 4:**
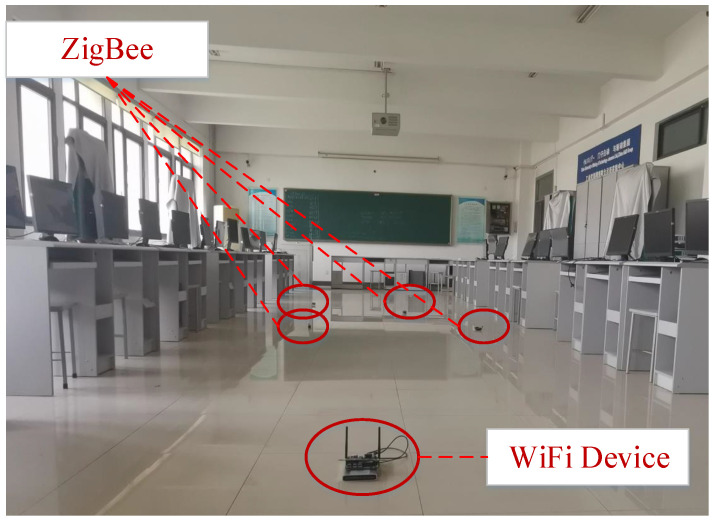
Experimental scene indoor.

**Figure 5:**
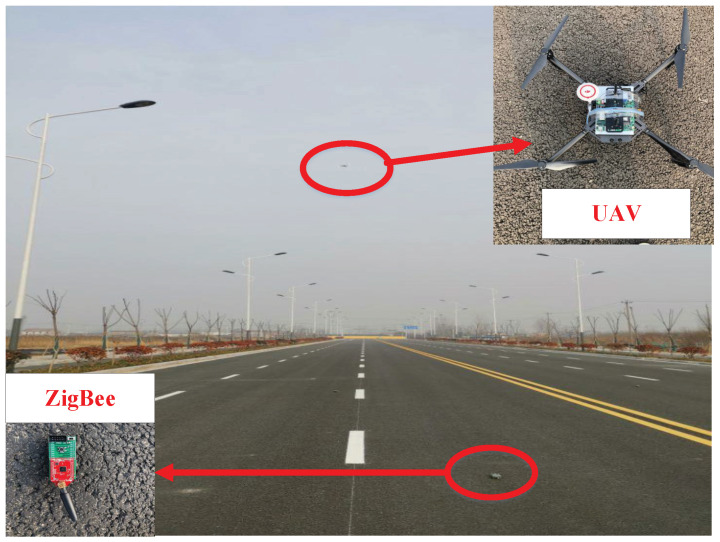
Experimental scene on the highway.

We implemented our algorithm with 30 low-power RF-CC2430 ZigBee nodes and a DJI M100 UAV on a 1 km highway and an indoor site, which are shown in Figure 4 and Figure 5. A UAV was used as the transmitting device and a microcomputer equip with an Atheros AR2425 wireless network card to send time synchronization packets. Moreover, 30 low-power RF-CC2430 ZigBee nodes were receivers, and each ZigBee node satisfied a sparse distribution. We selected an average value of 20 experiments as the experimental result to ensure the accuracy of the experiment.

### 5.2. Evaluation of Delay

In the experiment, the UAV as the transmitter only sends data packets and ZigBee as the receiver only receives data packets. The energy consumption of the ZigBee nodes is very low because ZigBee nodes are only charged with receiving the time synchronization data packet. Moreover, if we send the packet by using a UAV to carry the WiFi device, its transmission distance is not long. In such a short distances, any propagation delay can be ignored. we just pay attention to transmission delay and processing delay. Therefore, it is only necessary to measure the time synchronization accuracy at different transmission times to reflect the performance of the system. The total delay E(n) can be calculated as follows:

(14) $$E\left(n\right)=\left(D_{t}+D_{p}+T_{i}\right)\times \left(n-1\right)$$

where $D_{t}$ is transmission delay, which is determined by the packet length and bandwidth. $D_{p}$ is processing delay and $T_{i}$ is transmission time interval. *n* is re-transmission times. In our experiment, $D_{t}$ and $D_{p}$ are about 1.5 μs and 0.5 μs, respectively. $T_{i}$ is set to 8 μs. We can find that total delay can be determined by retransmission times *n*.

**Figure 6:**
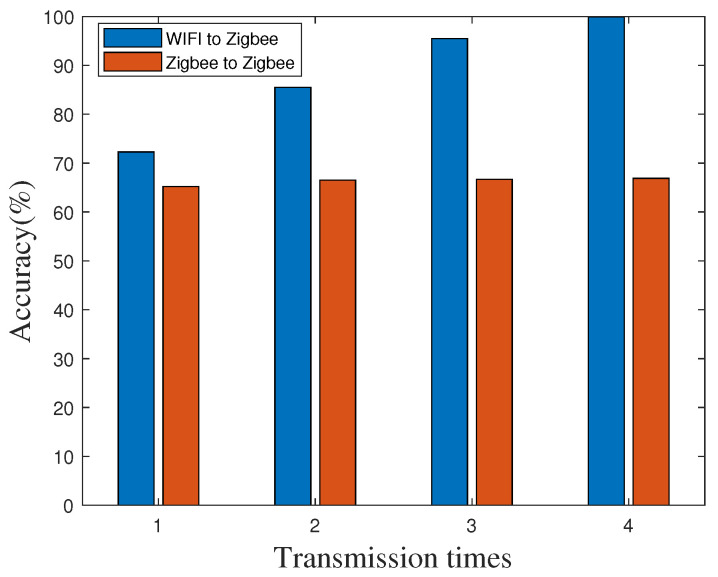
Time synchronization accuracy in different transmission times on the highway.

First, we measure the accuracy on the highway site by using the RF-CC2430 nodes as receivers and the WiFi device as the transmitter with various transmission times, and transmission distance is set to 20 m. The accuracy is the probability that the ZigBee node can accurately achieve time synchronization according to the synchronization data packet, which is mainly related to the packet reception rate. We measure and count the number of ZigBee nodes that can achieve time synchronization in different transmission times to calculate the accuracy in different transmission times. According to the accuracy and different transmission times, we draw the comparison histogram, as shown in Figure 6, the performance of “WiFi to ZigBee” is obviously better than that of “ZigBee to ZigBee”. The accuracy can achieve greater than 99% after 4 retransmission when using WiFi as the transmitter. Based on Formula (14), we can claim that time synchronization error is less than 30 μs in 99% of cases.

**Figure 7:**
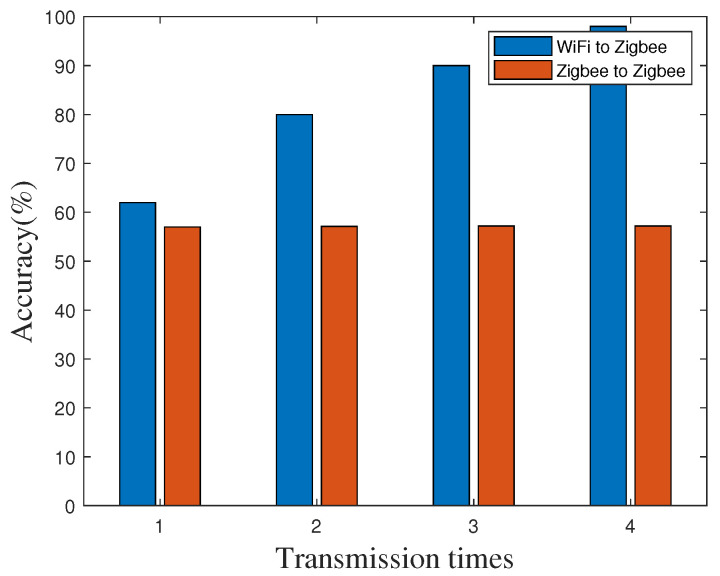
Time synchronization accuracy in different transmission times indoors.

At the same time, we measure the accuracy at an indoor site by using the RF-CC2430 nodes as receivers and the WiFi device as the transmitter with various transmission times. The transmission distance is also set to 20 m. As shown in Figure 7, because there are more obstacles and signal interference in the indoor environment, the performance of “WiFi to ZigBee” and “ZigBee to ZigBee” are lower than that in the outdoor site. But “WiFi to ZigBee” is still better than “ZigBee to ZigBee”. Based on Formula (14), we can claim that time synchronization error is less than 40 μs in 99% of cases.

### 5.3. Evaluation of Packet Reception Rate

**Figure 8:**
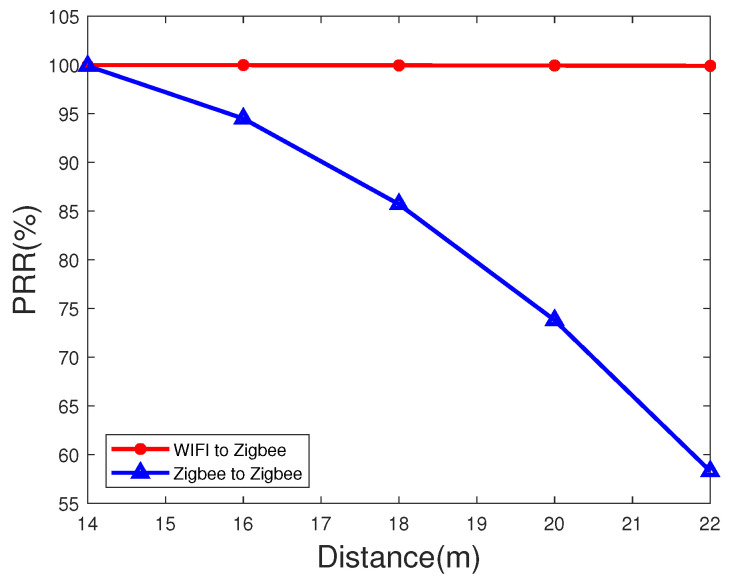
Packet reception rate at short distance on the highway.

**Figure 9:**
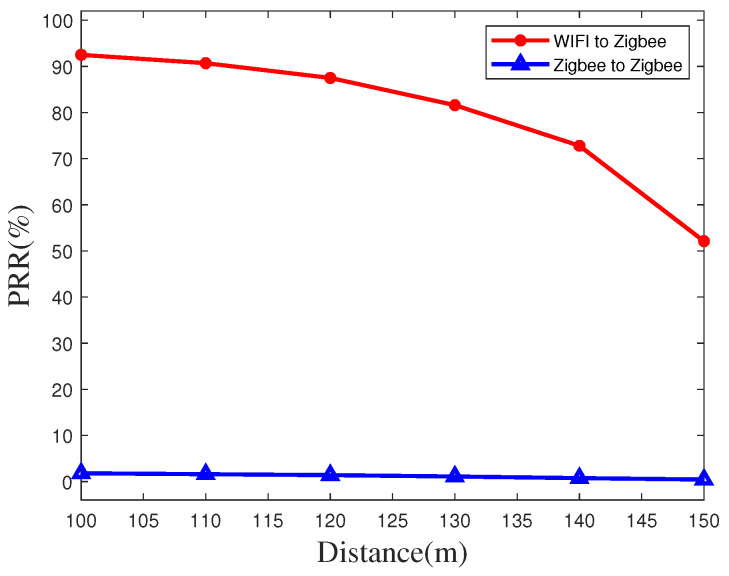
Packet reception rate at long distance on the highway.

We also implement the time synchronization packet reception rate (PRR) on the highway by using both the RF-CC2430 nodes and the WiFi as time synchronization transmitters at different transmission distances. We measure and count the number of ZigBee nodes that can achieve time synchronization in different transmission distances to calculate the accuracy in different transmission distances. According to the PRR and different transmission distances, we draw the comparison graphs as shown in Figure 8 and Figure 9. In the case of short distance, the PRR of our algorithm can reach 99%. As the distance increases, the reception rate of time synchronization packets gradually decreases. The ZigBee nodes exhibit larger reductions when used as transmitters. However, the PRR at a distance of 150 m can still reach 52.1% when using WiFi as a transmitter, which is much greater than the rate when using a ZigBee node as a transmitter.

**Figure 10:**
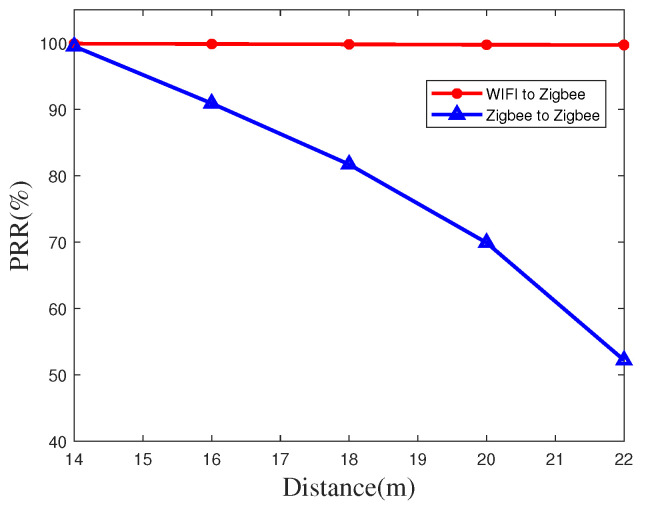
Packet reception rate at short distance indoors.

**Figure 11:**
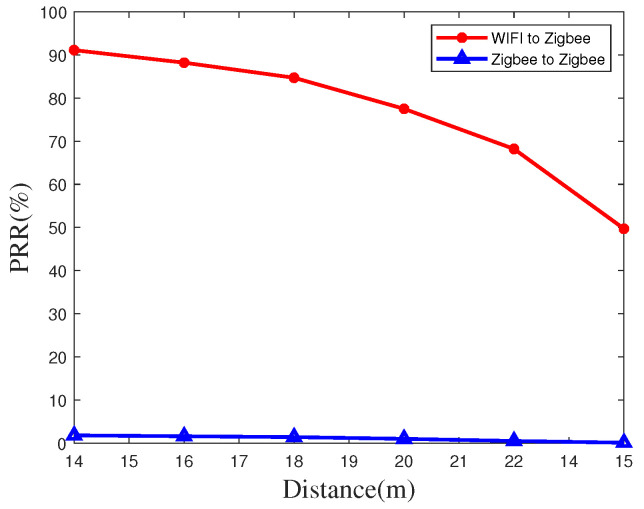
Packet reception rate at long distance indoors.

Similarly, we implement the time synchronization PRR at an indoor site by using the same devices at different transmission distances, as shown in Figure 10 and Figure 11. Due to the influence of the increase of interference, the value of PRR indoors will decreases, the PRR at a distance of 150 meters can still reach 49.8% when using WiFi as a transmitter, which is much greater than the rate when using a ZigBee node as a transmitter.

**Figure 12:**
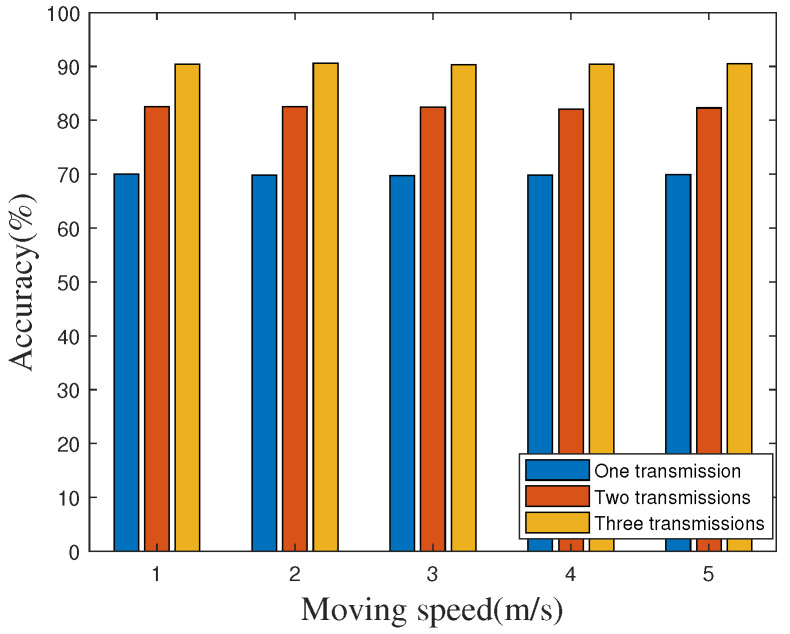
Effect of unmanned aerial vehicle (UAV) moving speed.

In a realistic time synchronization environment, the flying speed of the UAV is not stable, and various environmental factors such as wind power and environmental obstacles will affect the flying speed of the UAV. At the same time, different synchronization requirements may also affect the flying speed of the UAV. In order to verify whether the different flight speeds of the UAV will affect the time synchronization, we measure the accuracy on the highway by using the WiFi device as the transmitter at various speeds under the single transmission. As shown in Figure 12, although the speed of UAV is changing, the accuracy will almost not change. In other words, we can conclude that accuracy is not affected by the speed of UAV.

### 5.4. Evaluation of Energy Consumption

**Figure 13:**
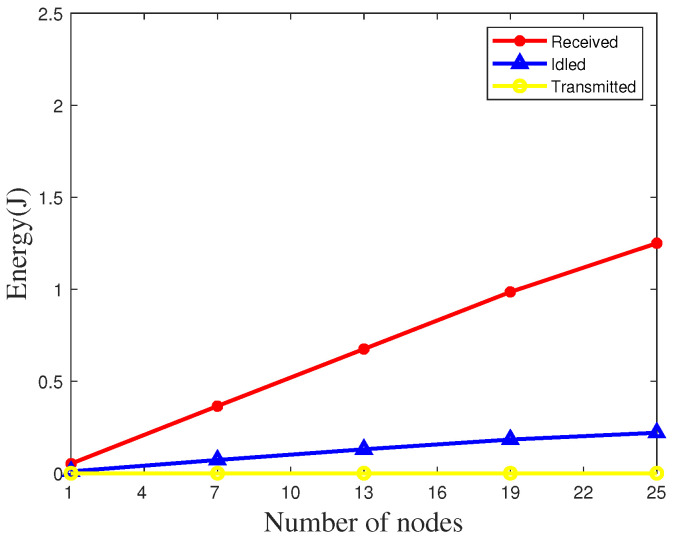
Evaluation of energy consumption on the highway.

**Figure 14:**
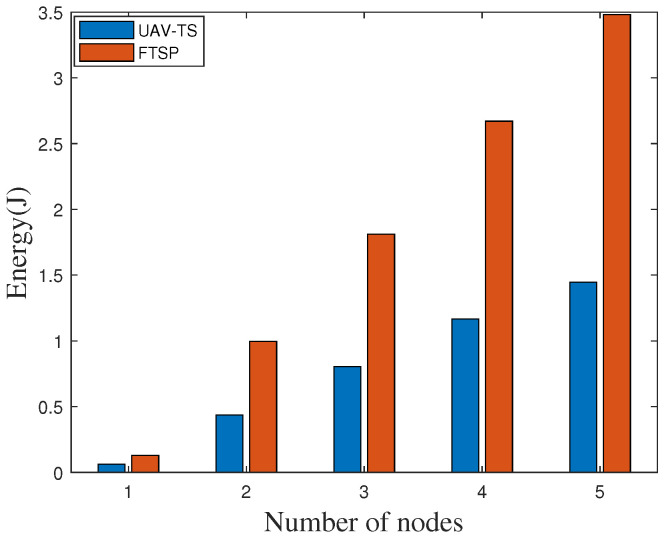
Comparison energy consumption with FTSP on the highway.

The energy consumption of ZigBee nodes in the network is mainly composed of idle energy consumption, transmitted energy consumption, and received energy consumption [48,49,50]. We measure the average energy consumption of the ZigBee nodes using our algorithm. The comparison of energy consumption of various parts under different numbers of nodes at an outdoor site is shown in Figure 13, since the node does not need to send data packets with our algorithm, only ZigBee nodes need to receive data packets, the energy consumption is extremely low, and the energy consumption of a single node is close to zero. At the same time, we compare the energy consumption of our algorithm with the widely used FTSP network method. As shown in Figure 14, whether it is a single node or multiple nodes, our algorithm can effectively reduce energy consumption.

**Figure 15:**
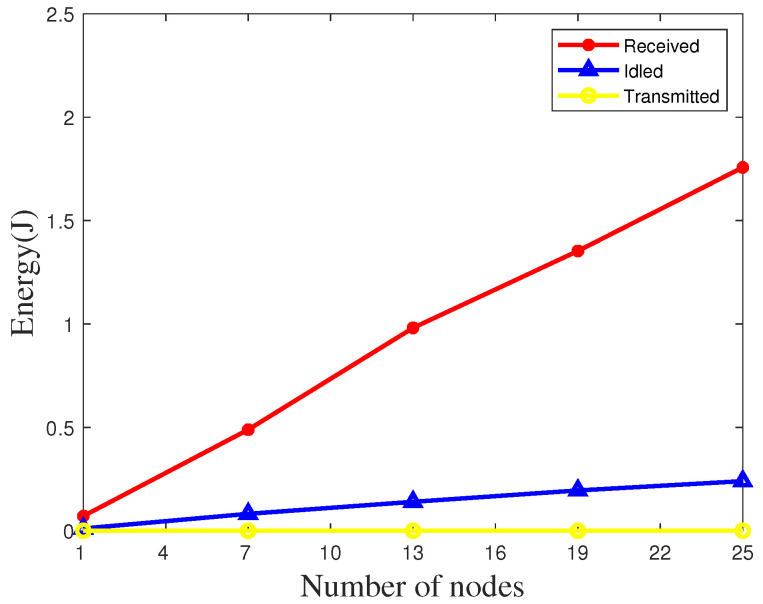
Evaluation of energy consumption indoors.

**Figure 16:**
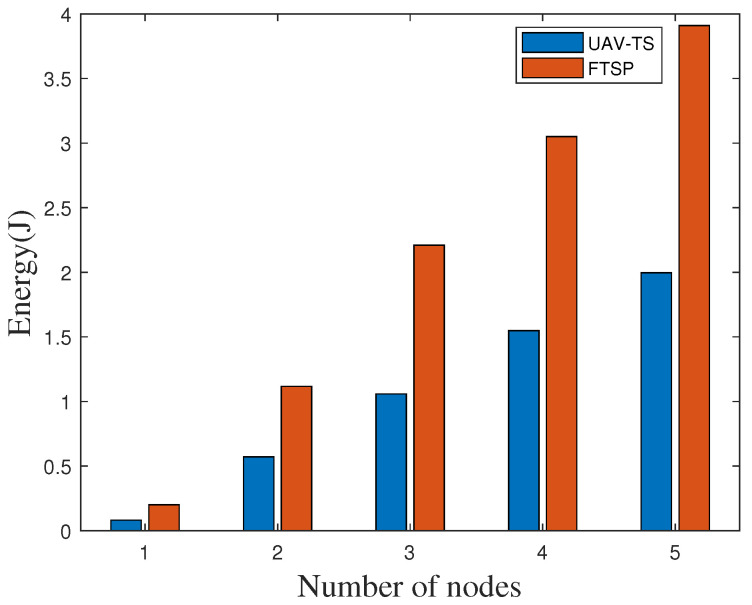
Comparison energy consumption with FTSP indoors.

We also measure the average energy consumption of the ZigBee nodes using our algorithm at an indoor site. The comparison of energy consumption of various parts under different numbers of nodes is shown in Figure 15. The energy consumption is still extremely low and the energy consumption of a single node is also close to zero. We also compare the energy consumption of our algorithm with the widely used FTSP network method. As shown in Figure 16, Due to the interference increases, both the received energy and idle energy of FTSP and UAV-TS increase. However, our algorithm can still effectively reduce energy consumption in the condition of a single node or multiple nodes.

## 6. Conclusions

To improve the time synchronization accuracy and reduce energy consumption for large-scale WSN, this paper proposes a novel UAV-assisted time synchronization algorithm based on cross-technology communication. This algorithm can accurately achieve time synchronization with almost zero energy consumption of nodes. We use 30 low-power RF-CC2430 transceivers with ZigBee nodes to perform experiments on a 1 km highway and an indoor site. The results show that time synchronization can be achieved accurately with almost no energy consumption for the sensor nodes, and the time synchronization error is less than 30 μs in 99% of cases on an 1 km highway.
